# The Ponseti method in children with clubfoot after walking age – Systematic review and metanalysis of observational studies

**DOI:** 10.1371/journal.pone.0207153

**Published:** 2018-11-20

**Authors:** Gabriel Ferraz Ferreira, Kelly Cristina Stéfani, Davi de Podestá Haje, Monica Paschoal Nogueira

**Affiliations:** 1 Foot and Ankle Surgery Group, Orthopaedics and Traumatology Unit, State Hospital of São Paulo, São Paulo, SP, Brazil; 2 Centro Clínico Orthopectus e Hospital de Base, Brasília, DF, Brazil; 3 Children’s Orthopaedics and Reconstruction Group, Orthopaedics and Traumatology Unit, State Hospital of São Paulo, São Paulo, SP, Brazil; University of California Los Angeles, UNITED STATES

## Abstract

**Background:**

The prevalence of untreated congenital clubfoot among children older than walking age is higher in developing countries due to limited resources for early care after birth. The Ponseti method represents an intervention option for older, untreated children.

**Methods:**

A metanalysis was conducted of observational studies selected through a systematic review of articles included in electronic databases (Medline, Scopus, Embase, Lilacs, and the Cochrane Library) until June 2017. A pooling analysis of proportions with 95% confidence intervals (CIs) and a publication bias assessment were performed as routine. Estimates of success, recurrence, and complication rates were weighted and pooled using the random effects model.

**Results:**

Twelve studies, including 654 feet diagnosed with congenital clubfoot in children older than walking age (older than 1 year old), were included for analysis. The rate of satisfactory outcomes found via a cluster metanalysis of proportions using the random effects model was 89% (95% CI = 0.82–0.94, p < 0.01), relative to the total analysed. The recurrence rate was 18% (95% CI = 0.14–0.24, p = 0.015), and the rate of casting complications was 7% (95% CI = 0.03–0.15, p = 0.19).

**Conclusion:**

Application of the Ponseti method in children with untreated idiopathic clubfoot older than walking age leads to satisfactory outcomes, has a low cost, and avoids surgical procedures likely to cause complications. The results obtained exhibited considerable heterogeneity.

## Introduction

Congenital clubfoot (CC) is a complex deformity. Conservative treatment, involving serial cast changes, is a consensus in the literature, with the Ponseti method considered to be the first choice [1]. However, this method is described for children younger than walking age, i.e., with diagnosis and treatment starting at birth.

For treatment of patients diagnosed with CC after walking age, most studies indicate extensive surgical release of the foot tissues with or without osteotomy [2].

However, in such cases, surgery is complex and often associated with serious complications and difficulty obtaining satisfactory outcomes [3]. In addition, surgery does not prevent recurrence, the rate of which is approximately 25%, and reoperation is frequently required, with a consequent increase in complications and limitations in functional outcomes [4,5]. Given this scenario and considering its success among younger children, the Ponseti method was indicated as a therapeutic option for older children with CC, i.e., older than 1 year old, being associated with low complication rates and lower cost [6].

The results of the Ponseti method in CC patients older than walking age (older than 1 year old) not previously treated were assessed as to the (1) rate of success in correcting the deformity, (2) rate of recurrence after the end of correction, and (3) incidence of complications.

## Materials and methods

### Search strategy

A systematic review was performed by two reviewers (G.F.F. and K.C.S.) according to the Preferred Reporting Items for Systematic Reviews and Meta-Analyses (PRISMA) guidelines [7]. Studies were located by searching the databases Medline (Pubmed), Cochrane Library, Lilacs, Scopus, and Embase. The search was performed on 3 June 2017 with the keywords “clubfoot” AND “Ponseti”, without any language restriction or filter. In addition, a manual search was performed of the references cited in studies, letters, reviews, and paediatric foot and ankle reference textbooks. The present systematic review was registered in the International Prospective Register of Systematic Reviews (PROSPERO) [8] under registration number CRD42017069054. Two reviewers (G.F.F. and K.C.S.) retrieved the data and independently analysed each selected study; instances of disagreement were resolved by the senior investigator (M.P.N.).

### Criteria for inclusion and exclusion

The inclusion criteria were (1) clinical diagnosis of idiopathic CC; (2) no previous treatment; (3) children after walking age at the onset of treatment (older than 1 year old); and (4) treatment using the Ponseti method. The exclusion criteria were (1) patients with CC due to neuromuscular disease or other specific cause; and (2) history of previous surgical manipulation before treatment. Response letters, comments, case reports, systematic reviews, and meta-analyses were excluded.

### Data extraction

Two reviewers independently extracted the data from the articles according to the following predefined criteria: name of first author, publication year, country, study design, type of study, number of patients, number of feet, age, and duration of follow-up. In addition, we collected data on the surgical procedures performed, recurrence, and differences in the performance of the Ponseti method among studies (Table 1).

**Table 1:** Summary of studies included in the systematic review.

Study	Publication year	Country	Study design	Type of study	Boys	Girls	Patients	Feet	Bilateral	Age in years mean (range)*
Mehtani et al.	2018	India	Case series	Prospective	23	18	41	73	51.2%	3.1 (1.1–12)
Sinha et al.	2016	India	Case series	Prospective	24	6	30	41	36.7%	3.02 (1–10.3)
Faizan et al.	2014	India	Case series	Prospective	16	3	19	28	47.4%	2.7 (1–3.5)
Ayana et al.	2014	Ethiopia	Case series	Prospective	17	5	22	32	50%	4.4 (2–10)
Qureshi et al.	2013	Pakistan	Case series	Prospective	29	21	50	N/S	N/S	1.64
Hassan et al.	2013	Egypt	Case series	Prospective	14	6	20	30	50%	1.59 (1–3)
Banskota et al.	2013	Nepal	Case series	Retrospective	19	17	36	55	52.8%	7.4 (5–10)
Verma et al.	2012	India	Case series	Prospective	30	7	37	55	48.6%	2.06 (1–3)
Yagmurlu et al.	2011	Turkey	Case series	Prospective	22	5	27	31	14.8%	1.76 (1–6)
Khan et al.	2010	India	Case series	Prospective	15	6	21	25	19%	8.9 (7.5–11.1)
Spiegel et al.	2009	Nepal	Case series	Retrospective	120	51	171	260	73.1%	N/S
Lourenco et al.	2007	Brazil	Case series	Retrospective	12	5	17	24	N/S	3.9 (1.2–9.0)

N/S = not specified

*Age at start of treatment with the Ponseti method

### Quality assessment

Following the hierarchy of methodological quality, we chose to analyse comparative studies with the highest quality (randomised trials), in which one of the arms corresponded to treatment of CC using the Ponseti method. However, given the lack of this type of study, we considered observational studies of patients treated with this method. For inclusion in the systematic review, the study had to have assessed the patients before and after treatment using the Ponseti method. We chose to use the methodological index for non-randomised studies (MINORS) [9] to assess the methodological quality of the selected observational studies. Studies with a score equal to or less than 11 were rated as low quality, and studies with a score equal to or more than 12 as high quality.

### Statistical analysis

A metanalysis of proportions was performed with data normalised using the logit function based on the selected dichotomous outcomes. Heterogeneity among studies was calculated using the I^2^ and τ^2^ statistics; random effects were considered in the present study. The results were described with the corresponding 95% confidence interval (95% CI). The significance level was set to p < 0.05. The random effects model was selected for the metanalysis, and the calculations were performed using the software R [10]. Subgroup and meta-regression analyses were performed to investigate the cause of the heterogeneity found. Publication bias was assessed using funnel plots and Begg’s test, where p < 0.05 indicated probable publication bias.

### Data items

The results gathered from the various databases were synthesised and categorised using EndNote X7.7.1 (Thomson Reuters, CA, USA). Duplicates were removed.

### Outcomes and prioritisation

The outcomes selected for assessment were the Pirani [11] score, Dimeglio [12] score, radiological assessment, final ankle dorsiflexion, rate of success, rate of recurrence, and complications associated with the Ponseti method. Success was defined as a pain-free, aesthetically acceptable plantigrade foot, with no need for extensive surgical tissue release after casting and tenotomy.

## Results

### Eligible studies

**Fig 1:**
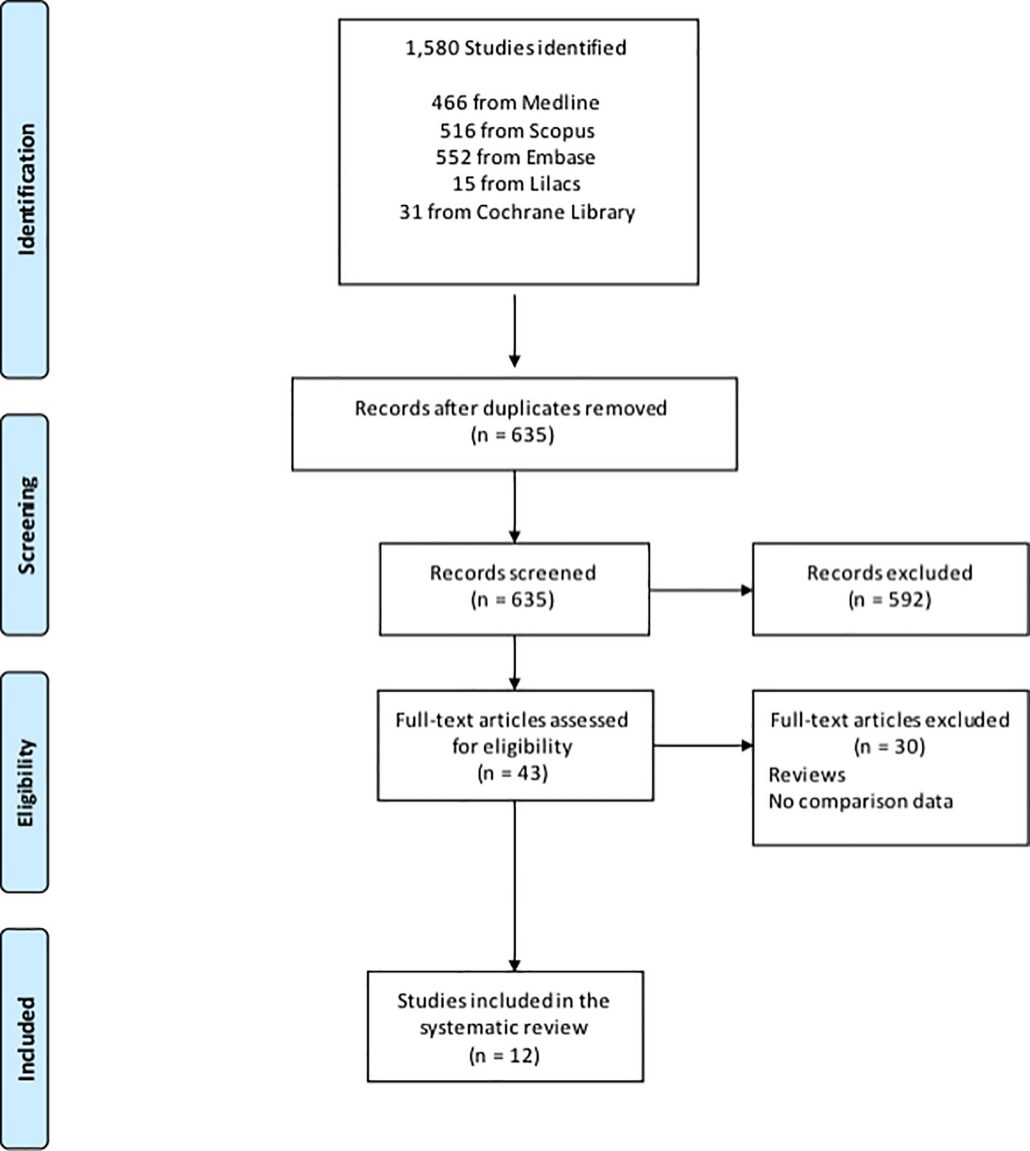
PRISMA flowchart of the literature search and study selection.

The search conducted using the aforementioned keywords retrieved 1,580 articles: 466 from Medline (PubMed), 516 from Scopus, 552 from Embase, 31 from the Cochrane Library, and 15 from Lilacs. After the exclusion of duplicates and irrelevant articles, 43 studies were carefully analysed by the authors. Finally, 12 observational studies that met the established criteria were selected for the metanalysis. The flowchart representing study selection is shown in Fig 1.

### Demographic characteristics of the included studies

The main characteristics of the selected studies are described in Table 1. The studies were published from 2007 to 2017. We did not find any prospective controlled studies. A total of 12 case series were identified: five from India [13–17], two from Nepal [18,19], and the remainder from Brazil [6], Turkey [20], Egypt [21], Pakistan [22], and Ethiopia [23]. In total, the studies assessed 491 patients, including 341 boys and 150 girls, aged 1 to 11 years old.

The cases included in the studies were evaluated based on objective scores and clinical and radiological assessment. For the calculation of rates, success was defined as an aesthetically acceptable plantigrade foot, without any residual deformity or extensive surgical release of the tissues at the end of the initial correction (Table 2).

**Table 2:** Clinical and radiological assessment before and after treatment using the Ponseti method.

Study	Pirani baseline mean (range)	Pirani final mean (range)	Dimeglio baseline mean (range)	Dimeglio final mean (range)	Ankle dorsiflexion mean (range)*	Radiological assessment baseline mean	Radiological assessment final mean	Follow-up in years mean (range)	Success rate** feet (%)
Mehtani et al.	4.21	0.03	15.9	0.52	21.3 (10–45)	N/S	N/S	3.0 (1.2–4)	69 (94%)
Sinha et al.	5.41	0.12	15.9	2.07	1–3 years old: 15.48 3–5 years old: 8.54 > 5 years old: 7	Anteroposterior talocalcaneal = 5.04° Lateral talocalcaneal = 5.29° Beatson-Pearson index = 10.34°	Anteroposterior talocalcaneal = 26.14° Lateral talocalcaneal = 25.24° Beatson-Pearson index = 51.39°	2.6 (2–3.9)	41 (100%)
Faizan et al.	4.84 (3.5–5.5)	0.55 (0–1)	12.96 (10–14)	2.32 (2–3)	16 (13–24)	N/S	N/S	2.7 (1.5–3.5)	26 (92.8%)
Ayana et al.	5	0	N/S	N/S	N/S	N/S	N/S	3 (2–4)	32 (100%)
Qureshi et al.	N/S	1.31 (+/- 0.43)	N/S	N/S	N/S	N/S	N/S	No follow-up	34 patients (68%)
Hassan et al.	4.85 (2–6)	0.5 (0–1)	N/S	N/S	12.5	N/S	N/S	2.5 (2–4)	30 (100%)
Banskota et al.	5.1 (3–6)	2.1 (1.5–4)	15.9 (11–20)	5.9 (4–14)	9 (0–15)	N/S	N/S	2.62 (2–3.34)	46 (84%)
Verma et al.	4.95 (3.5–6)	0.76	N/S	N/S	11.9	N/S	N/S	2.5 (1.25–3)	49 (89.1%)
Yagmurlu et al.	N/S	N/S	Grade 3 = 96.7% Grade 4 = 3.3%	Grade 1 = 87.0% Grade 2 = 13.0%	N/S	N/S	N/S	2.6 (2.16–3.16)	31 (100%)
Khan et al.	N/S	N/S	14.2	0.18	7 (5–10)	Beatson-Pearson index < 8°	Beatson-Pearson index = 55° (45° - 65°)	4.7	18 (85.7%)
Spiegel et al.	5.15	2.07	N/S	N/S	12.49	N/S	N/S	No follow-up	246 (94%)
Lourenco et al.	(4–5)	N/S	N/S	N/S	5 (0–10)	Beatson-Pearson index < 10°	Beatson-Pearson index = 42° (37° - 62°) Talo-first metatarsal angle = 9.7° (6° - 12°)	3.1 (2.1–5.6)	16 (67%)

N/S = not specified

* Evaluated after the end of treatment

**Considered as a pain-free, deformity-free, aesthetically acceptable plantigrade foot with no need for the extensive release of soft tissues.

The quality of the studies was assessed using MINORS [9]. Studies with a score of 11 or less were rated as low quality, and studies with a score of 12 or above were rated as high quality. The summarized study quality assessment is shown in Table 3.

**Table 3:** Summary of the study quality assessment using minors (methodological index for non-randomised studies)*.

Study	1. A stated aim	2. Inclusion of consecutive patients	3. Prospective data collection	4. Endpoint appropriate to the study aim	5. Unbiased evaluation of endpoints	6. Follow-up period appropriate to the major endpoint	7. Loss to follow-up not exceeding 5%	8. Prospective calculation of sample size	Total score**	Study quality***
Mehtani et al.	2	0	2	2	0	2	0	1	9	Low
Sinha et al.	2	0	2	2	0	2	2	1	11	Low
Faizan et al.	2	0	2	2	0	2	0	1	9	Low
Ayana et al.	2	2	2	2	0	2	2	0	12	Low
Qureshi et al.	2	2	2	2	0	1	0	0	9	Low
Hassan et al.	2	0	2	2	0	2	0	0	8	Low
Banskota et al.	2	1	1	2	0	2	1	0	9	Low
Verma et al.	2	2	2	2	0	2	0	1	11	Low
Yagmurlu et al.	2	0	2	2	0	2	0	1	9	Low
Khan et al.	2	0	2	2	0	2	0	0	8	Low
Spiegel et al.	2	2	1	2	0	1	0	1	9	Low
Lourenco et al.	2	0	1	2	0	2	2	0	9	Low

*Methodological index for non-randomised studies (without additional criterion in the case of comparative studies)

**Recorded as 0 (non-reported), 1 (reported but inadequate), or 2 (reported and adequate)

*** Studies with a total score equal to or above 12 were rated as having a high methodological quality.

There were slight variations in the application of the Ponseti method among the studies. Variations concerned the intervals between cast changes, type of brace used after the casting phase, and duration of immobilization after Achilles tenotomy, among others, as described in Table 4.

**Table 4:** Summary of variations in the Ponseti method and complications.

Study	Number of casts mean (range)	Time to cast change (weeks)	Orthosis type	Orthosis protocol	Duration of immobilisation with cast after Achilles tenotomy (weeks)	Ponseti method complications
Mehtani et al.	6.9 (4–10)	1	Abduction orthosis	< 5 years old = night sleep until age 4–5 years old > 5 years old = 3–6 months of use only	2 (changed every 2 weeks)	Superficial wounds and toe erythema and swelling
Sinha et al.	12.8 (8–18)	1	Abduction orthosis	All patients = 23 h for 3 months < 4 years old = night sleep until 4 years old > 4 years old = night sleep for 1 year	3	Erythema and superficial wounds
Faizan et al.	8 (5–12)	1	Abduction orthosis	Continuous use for 23 hours for 3 months	3	Superficial wounds
Ayana et al.	8 (6–10)	2	> 4 years old = AFO < 4 years old = AO	> 4 years old = AFO for 1 year < 4 years old = AO for 3 months (24 h) then at night for 9 months	4	N/S
Qureshi et al.	Maximum of 9 casts	1	N/S	N/S	3	N/S
Hassan et al.	6 (4–8)	2	Abduction orthosis	Until age 5	3	N/S
Banskota et al.	9.5 (6–11)	5 to 7 days	Ankle and foot orthosis	Use at night for at least 1 year	6	N/S
Verma et al.	10 (6–12)	1	Abduction orthosis	3 months	3	None
Yagmurlu et al.	6	7 to 8 days	Abduction orthosis	3 months	N/S	N/S
Khan et al.	12.1 (10–14)	1	Pronation shoes	2 years	4	Superficial wounds
Spiegel et al.	7	5 days	Abduction orthosis	Use at night until age 5	3	N/S
Lourenco et al.	9 (7–12)	2	Ankle and foot orthosis	12 months	5	Superficial wounds, toe erythema and swelling, and immobilisation-induced osteopaenia

N/S = not specified; AFO = ankle and foot orthosis; AO = abduction orthosis

The need for other surgical treatments in combination with the Ponseti method and treatment failure are described in Table 5. Failure of treatment using the Ponseti method was defined as the need for extensive surgical release with or without combined procedures involving bones.

**Table 5:** Summary of the surgical procedures performed.

Study	Open Achilles tendon lengthening	Percutaneous Achilles tendon lengthening	Anaesthesia for Achilles tenotomy	Tibialis anterior tendon transfer	Extensive release of soft tissues or bone procedure*	Posterior release	Posteromedial release	Surgical complications
Mehtani et al.	N/S	All feet	Local	4	Not performed	N/S	N/S	None
Sinha et al.	Not performed	All feet	General or local	3	Not performed	N/S	N/S	None
Faizan et al.	1 (3.6%)	All feet	Local	1 (3.6%)	Not performed	Not performed	Not performed	None
Ayana et al.	7	21	N/S	1	Not performed	4	Not performed	None
Qureshi et al.	N/S	All feet	N/S	N/S	N/S	N/S	N/S	None
Hassan et al.	N/S	21 (70%)	Local	4	N/S	N/S	2	N/S
Banskota et al.	27 (49%)**		Local	Not performed	1 (2%)	19 (34.5%)	8 (14.5%)	Superficial wound infection (7 feet, 12.6%) Deep wound infection (1 foot, 1.8%)
Verma et al.	3	44	Local	4	Not performed	1	6	None
Yagmurlu et al.	17	14	N/S	N/S	Not performed	N/S	N/S	N/S
Khan et al.	N/S	All feet	General or local	N/S	1 (4%)	N/S	6	N/S
Spiegel et al.	8 (3%)	205 (79%)	General or local	N/S	16 (6%)	21 (8%)	16 (6%)	Wound dehiscence (5 cases)
Lourenco et al.	15	All feet	Local	Not performed	Not performed	8	Not performed	None

N/S = not specified

*Considered as Ponseti method treatment failure

**Data presented for both open tenotomy and percutaneous tenotomy

Recurrence was reported in most studies, occurring at variable intervals throughout the follow-up. Most studies chose to repeat casting according to the Ponseti method with or without surgical procedures (Table 6).

**Table 6:** Summary of therapeutic options after the diagnosis of recurrence.

Study	Recurrence (feet)	Casting after recurrence	Second tenotomy	Dynamic supination	Comments
Mehtani et al.	8 (10.6%)	Yes	Yes	6	3 feet required tibialis anterior tendon transfer 5 feet were corrected after the second casting series with or without a second Achilles tenotomy
Sinha et al.	7 (17%)	Yes	Yes	N/S	3 feet had equinus deformity relapse 4 feet had equinus, cavus, and adduction deformity recurrence All cases underwent an additional casting series 4 feet were subjected to tenotomy and 3 to tibialis anterior tendon transfer
Faizan et al.	2 (7.2%)	Yes	Yes	1	1 foot with equinus deformity recurrence was treated with Achilles tendon lengthening 1 foot with dynamic supination was treated with tibialis anterior tendon transfer
Ayana et al.	4 (12.5%)	Yes	Yes	1	2 feet had a second casting series and tibialis anterior tendon transfer 2 feet had a second casting series and Achilles tendon lengthening with posterior capsulotomy
Qureshi et al.	Did not describe follow-up after the deformities were corrected				
Hassan et al.	6 (20%)	Yes	No	4	2 feet with equinovarus and adduction deformities were treated with 3 cast changes followed by medial release, abductor tenotomy, second Achilles tenotomy, and tibialis anterior tendon transfer 4 feet with dynamic supination were treated with a second casting series and tibialis anterior tendon transfer
Banskota et al.	9 (16%)	No	Yes	N/S	4 feet underwent Achilles tendon lengthening 4 feet underwent a posterior release of soft tissues 1 foot underwent a posteromedial release
Verma et al.	15 (27%)	Yes	Yes	4	7 one-sided feet developed a recurrence of forefoot adduction, hindfoot varus, and equinus deformity 5 feet developed a recurrence of equinus deformity alone 4 patients exhibited dynamic supination and were subjected to tibialis anterior tendon transfer
Yagmurlu et al.	0	No	No	0	No recurrence observed
Khan et al.	6 (24%)	No	No	N/S	4 cases underwent a posteromedial release of soft tissues 1 case underwent a lateral column shortening combined with an extensive release of soft tissues
Spiegel et al.	Did not describe follow-up after the deformity was corrected				
Lourenco et al.	7 (29%)	N/S	Yes	4	4 feet with dynamic supination, which did not impair gait; the decision was not to perform tibialis posterior tendon transfer

N/S = not specified

### Pooled analysis

The outcomes success, recurrence, and complication rates yielded results that made a pooled analysis possible.

### Success rate

**Fig 2:**
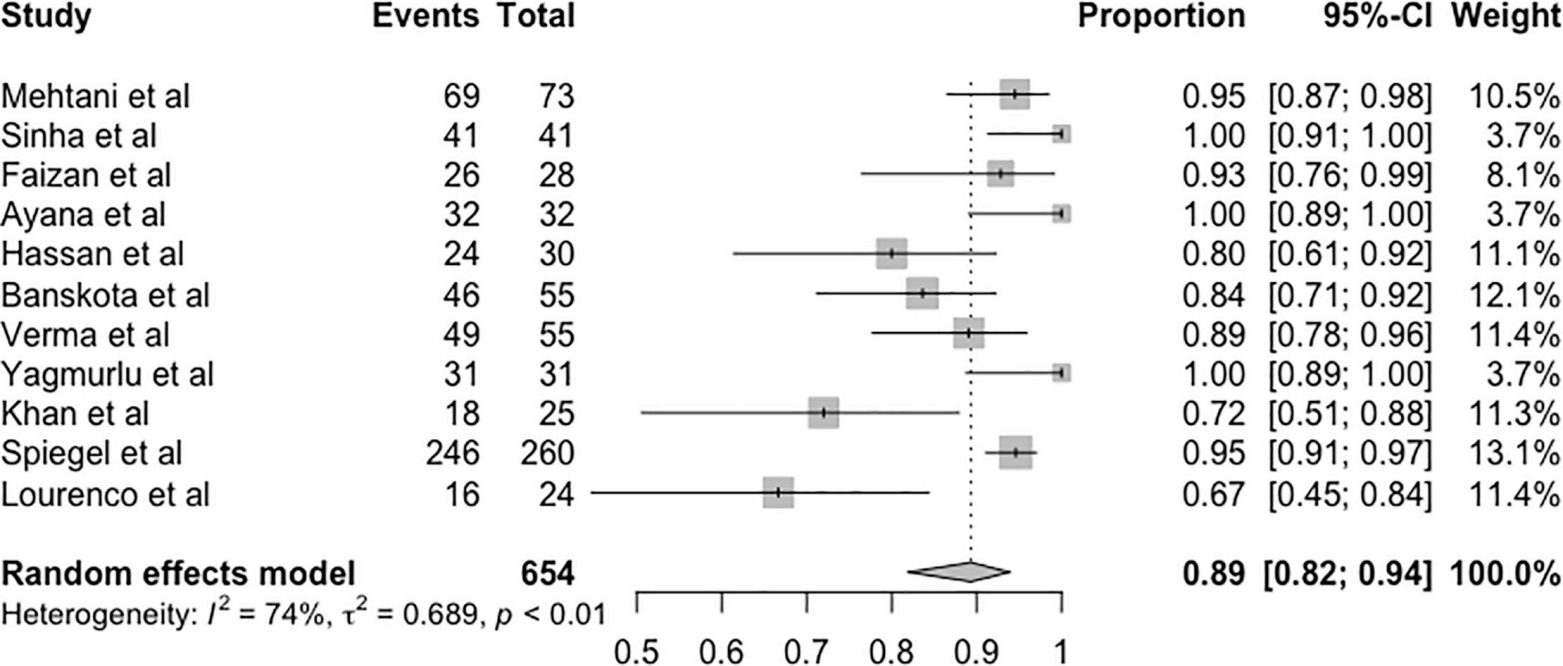
Forest plot of the metanalysis of studies examining the effect of treatment success.

In the analysis of treatment success—defined as satisfactory outcomes at the end of treatment—among the 12 included studies, only Qureshi et al. [22] was not included in the analysis because the success rate was calculated per the number of patients rather than per the total number of feet, which was different from the other studies. According to the random effects model, the success rate was 0.89 (95% CI = 0.82–0.94, p < 0.01) relative to the total analysed (Fig 2).

There was a high degree of heterogeneity among the results (I^2^ = 74%). Therefore, a subgroup metanalysis and meta-regression analysis were performed to investigate the source of heterogeneity. In the analysis stratified by age, the rate of success according to the random effects model for the group of children over 3 years old was 87.41% (95% CI = 0.7341–0.9458; Q = 19.62; τ^2^ = 0.8531; I^2^ = 74.5%), and it was 88.84% (95% CI = 0.7885–0.9445; Q = 4.71; τ^2^ = 0.2116; I^2^ = 36.3%) for the group of children under 3 years old. There was not a significant linear association between the final outcome and the mean age of patients (p = 0.8225) (S1 Appendix e S2 Appendix).

### Recurrence

**Fig 3:**
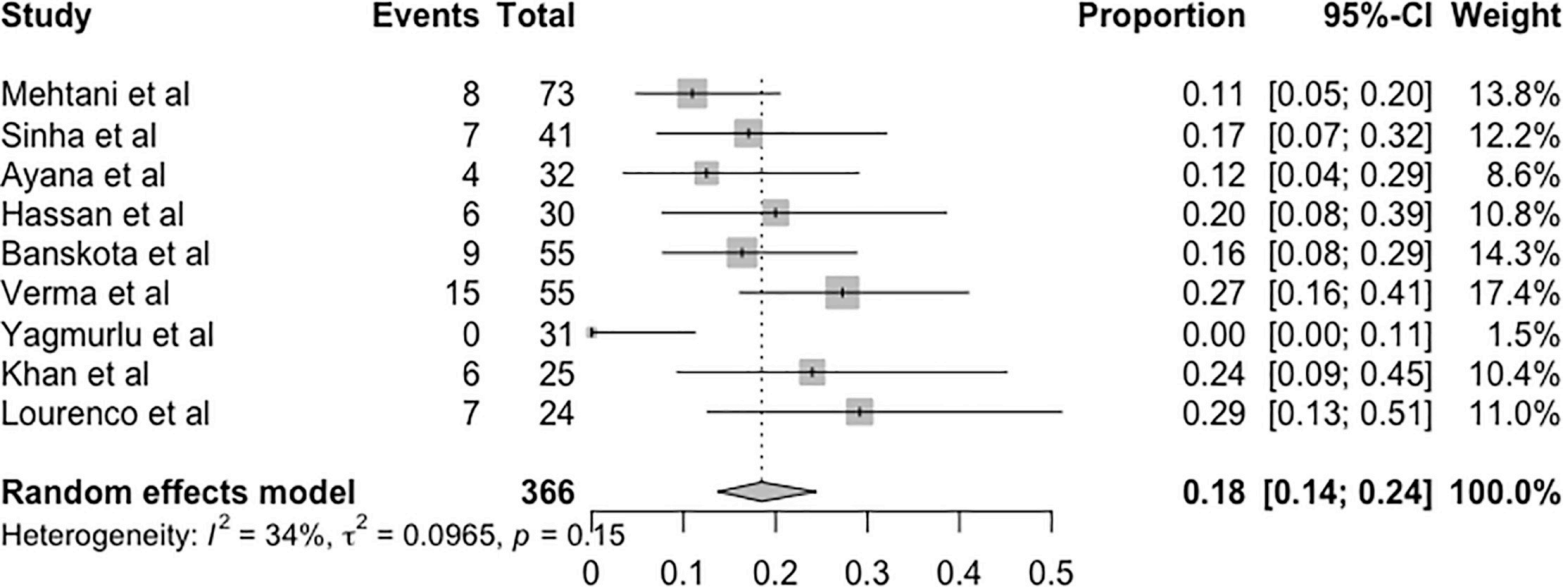
Forest plot of the metanalysis of studies examining the effect of recurrence after treatment.

Three studies [15,18,22] were excluded from the analysis of the recurrence rate of deformity after the end of treatment because they did not provide information on follow-up or the follow-up was not clearly described. The proportion of recurrence after the end of treatment using the Ponseti method was 0.18 (95% CI = 0.14–0.24, p = 0.15) according to the random effects model (Fig 3).

### Casting complications

**Fig 4:**
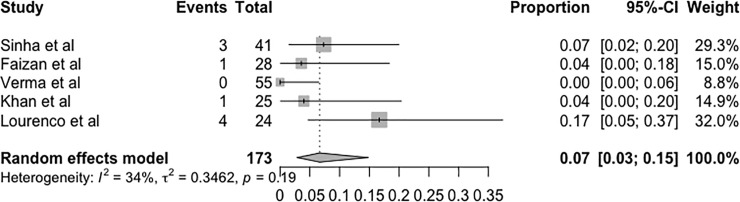
Forest plot of the metanalysis of studies examining the effect of casting complications.

The development of complications associated with Ponseti casting was dichotomously categorised as present or absent and described as a local wound, swelling, erythema and osteopenia. Only five studies were included [6,13,15–17] in this metanalysis because they were the only studies that described in detail the number of complications. According to the random effects model, the proportion of complications was 7% (95% CI = 0.03–0.15, p = 0.19) (Fig 4).

### Publication bias

**Fig 5:**
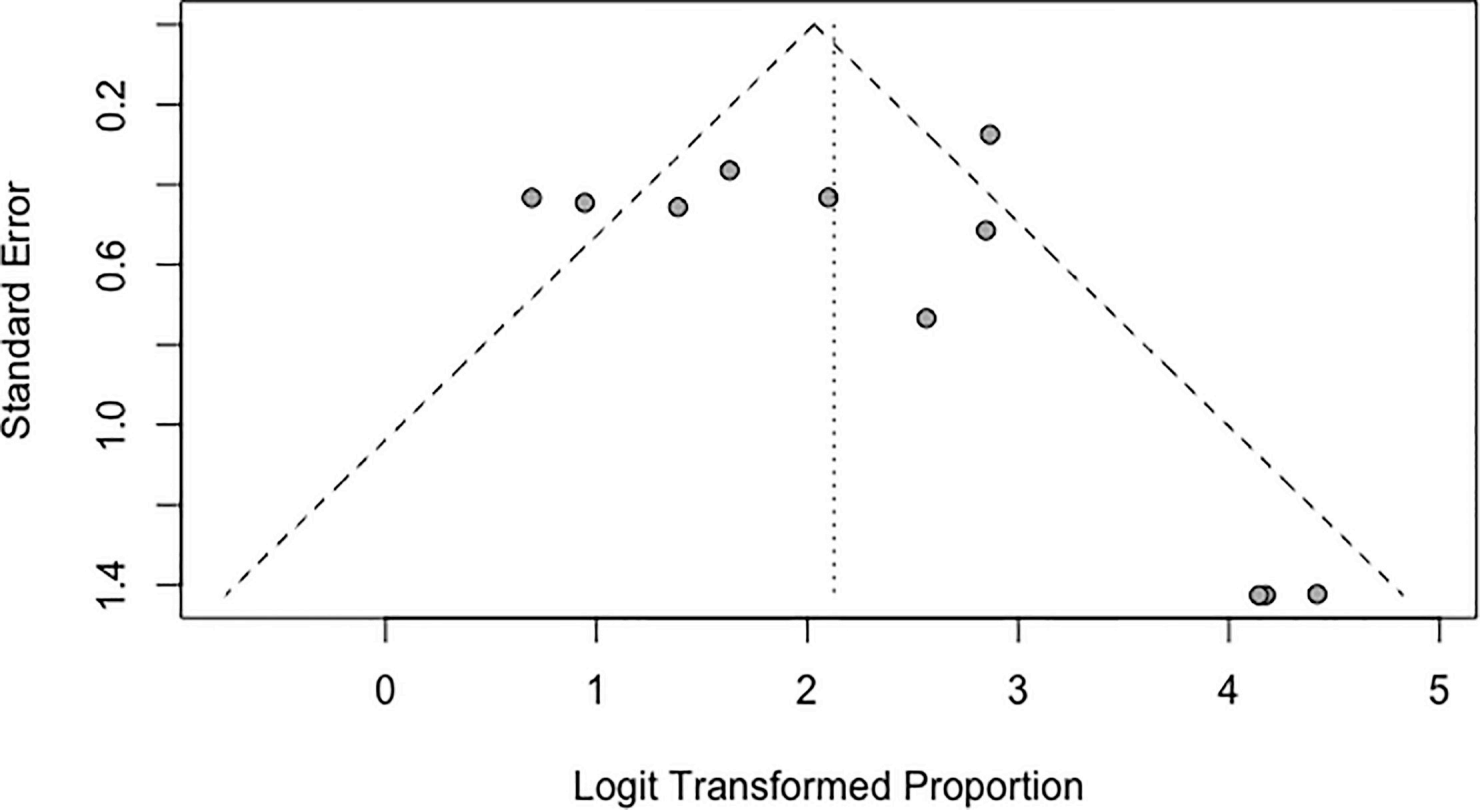
Funnel plot of the publication bias of studies included in the systematic review.

Begg’s test was used to investigate the occurrence of publication bias. No evidence of publication bias was found for the main outcome, satisfactory results (p = 0.3918). Despite the presence of two asymmetric studies, the funnel plot was homogeneous and thus indicative of the unlikeliness of publication bias (Fig 5).

## Discussion

Most cases of untreated CC occur in developing countries, due to deficiencies in the local public health systems, whereby children are not adequately treated [24]. The deformity becomes worse when walking because the body weight falls on the lateral side of the foot, causing contracture of the medial tissues and plastic deformity of the bones [25].

The Ponseti method was originally developed for children aged up to six months old, with a reproducibility of extremely high success rates [26]. One usual treatment for children with CC older than walking age is comprehensive surgery, with extensive posteromedial release being the procedure most frequently performed [3]. Other options include tarsectomy, midfoot osteotomy, arthrodesis, and correction with a circular external fixator, among others [27–29]. This type of treatment demands commitment, the postoperative period is long [24], and the rate of complications and cost are high [30]. In addition, the result is often a painful foot in childhood [31].

Furthermore, the skin on the affected feet develops ulcers or hygromas, which make surgical incisions difficult [25]. Dobbs et al. [31] reported poor results for approximately 50% of patients treated with the extensive surgical release of soft tissues after a 25-year follow-up, with stiffness being the main complication.

There are definite variations among studies in the application of the Ponseti method to patients after walking age. A large portion of the studies included in the present systematic review set one-week intervals between cast changes and three weeks of casting after tenotomy, which was most often performed under local anaesthesia. Abduction orthosis was the most frequent type of brace used after the end of correction.

Mehtani et al. [14] went beyond simple variations and truly modified the Ponseti method: they reapplied the post-tenotomy cast at two weeks in the maximum achievable dorsiflexion and abduction. In addition, they switched to below-knee, weight-bearing casts in maximum dorsiflexion and external rotation after removal of the post-tenotomy cast. According to these authors, these modifications allowed achievement of better final ankle dorsiflexion, which is considered a movement of crucial relevance. These modifications were considered occasional, and the Ponseti method was applied and maintained in essence; thus, the study was included in the results of the outcomes of the meta-analysis.

The rate of casting complications was low in the cluster analysis of studies (approximately 7%). The most common complications were erythema and superficial abrasions due to the low complexity and minimal degree of invasiveness of the Ponseti method.

The results of the Ponseti method for the treatment of children older than walking age reported in the studies included in the present systematic review and metanalysis are encouraging. Success was achieved in approximately 89% of the treated feet (Fig 3), i.e., aesthetically acceptable, functional, and pain-free plantigrade feet with no need for osteotomy or the extensive release of soft tissues.

The objective Pirani [11] and Dimeglio [12] scores are other relevant criteria to assess the final outcome of treatment. These scales were used in most studies to compare deformity before and after the end of treatment.

However, although treatment with the Ponseti method resulted in functional feet, recurrence occurred, with the partial and even complete reappearance of deformity. The recurrence rate obtained in the metanalysis of proportions was 18%. Recurrence might represent one of the main objections to the application of the Ponseti method to older children. Data show that such rate could be reduced through the correct use of abduction brace.

Recurrence may be explained by the fact that older children had greater deformities in the feet and less elasticity [32]. However, a recent study demonstrated the remodelling capacity during the application of the Ponseti method in a child with CC after walking age [33], reinforcing its viability in this age group.

The studies showed variations in the patients' ages, and it was inferred that differences in skeletal maturity existed at the time of treatment initiation. In addition, the follow-up time was short in most studies, which may affect the recurrence rate because it is believed that a longer follow-up time results in a greater number of recurrent cases.

Non-adherence to orthosis use following clubfoot correction may be considered a decisive factor for recurrence. Some studies emphasized the use of braces as crucial to avoid recurrence and called attention to the non-compliance of parents with the protocol, resulting in slight to severe recurrence of CC [31,34,35].

According to Khan et al. [17], three reasons account for the high rate of recurrence: ligament thickening, retraction of the tibialis posterior tendon, and low adherence to orthosis use after serial cast changes and Achilles tenotomy.

Approximately 60% of the studies that described the follow-up of cases treated recurrence with recasting. In general, the first sign of recurrence found in the studies was the loss of ankle dorsiflexion.

The present study has several limitations. First, the selected studies were case series, i.e., observational studies considered to have low methodological quality. Second, there were some slight variations in the application of the Ponseti method among the studies. Third, the follow-up time was limited in the studies, which may have altered the recurrence rate and the need for surgical correction. Lastly, the sample was heterogeneous among the studies regarding patient age, which may have altered the final outcomes and recurrence because variation in age represents a difference in skeletal maturity of the patients. These factors may influence the risk of bias and impair the value of the study conclusions.

## Conclusions

Application of the Ponseti method to patients with neglected idiopathic CC after walking age exhibited satisfactory outcomes (89%) and a low recurrence rate (18%) and allowed avoiding surgical procedures likely to cause complications.

There was high heterogeneity among the results of the analysed samples. Multicentre studies with uniform patient sampling methods, as well as uniformity in the application of the Ponseti method, are necessary to confirm the results obtained.

## Supporting information

S1 Appendix A subgroup metanalysis and meta-regression. The success rate from age stratified subgroup analysis (greater than or less than 3 years of age).
(TIFF)

S2 Appendix Bubble chart to compare the mean age of the patients and the success rate. There was not a significant linear association between the final outcome and the mean age of patients.
(TIFF)

S1 File PRISMA checklist. Checklist according to PRISMA protocol.
(DOC)
