# Supplementary figures and images for: The Ponseti method in children with clubfoot after walking age – Systematic review and metanalysis of observational studies
Source: PLoS One. 2018 Nov 20;13(11):e0207153. doi: 10.1371/journal.pone.0207153 (PMC6245511; doi:10.1371/journal.pone.0207153)

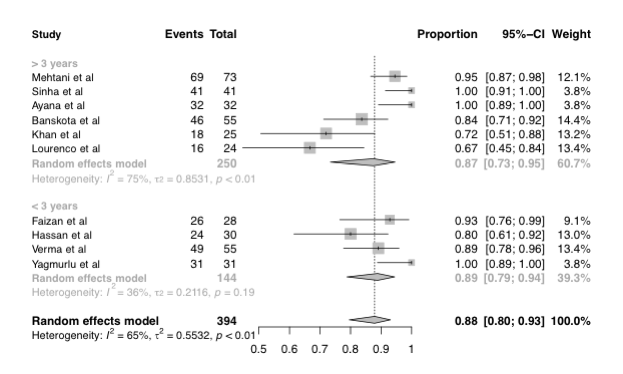

Supplement: S1 Appendix — The success rate from age stratified subgroup analysis (greater than or less than 3 years of age). (TIFF) [file pone.0207153.s001.tiff]

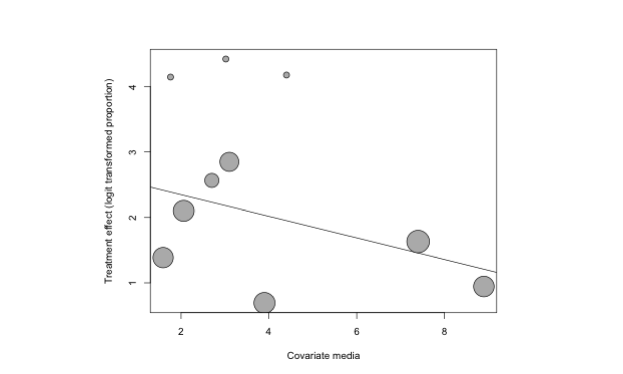

Supplement: S2 Appendix — There was not a significant linear association between the final outcome and the mean age of patients. (TIFF) [file pone.0207153.s002.tiff]
